# The neurophysiological effects of frontotemporal lobar degeneration: linking synaptic loss, network connectivity and cognitive decline

**DOI:** 10.1002/alz.087315

**Published:** 2025-01-03

**Authors:** James B Rowe

**Affiliations:** ^1^ Department of Clinical Neurosciences and Cambridge University Hospitals NHS Trust, University of Cambridge, Cambridge United Kingdom

## Abstract

**Background:**

Frontotemporal dementia (FTD) and Progressive Supranuclear Palsy (PSP) have distinct molecular pathologies, with Tau and TDP43 aggregation, and distinct patterns of regional brain atrophy. However, they share the synaptotoxicity of protein aggregation, and neurotransmitter loss (including GABA), which contribute to clinical and neurophysiological similarities. Defining the relationships between synaptic loss, network physiology and cognition builds bridges between preclinical and clinical studies, and facilitates early phase trials.

**Method:**

We quantified the effect of behavioural variant frontotemporal dementia (±parkinsonism) and progressive supranuclear palsy (Richardson’s syndrome, and PSP‐F), and controls with Magnetoencephalography (resting state, mismatch task, and motor control task); 11‐C‐UCBJ PET estimation of synaptic density; 3T T1w and fMRI.

**Result:**

Participants with bvFTD showed severe synaptic loss compared to controls which correlated strongly with baseline cognitive function (ACE‐r r∼0.8, p<0.001). Participants with PSP showed severe synaptic loss, which progressed over 12 months; the degree of synaptic loss over prefrontal cortex correlated with functional decline (PSPRS, r∼0.47, p<0.03. In both PSP and FTD, synaptic loss was more severe and widespread than cortical atrophy; prefrontal/ACC atrophy was significant in PSP, but less than in bvFTD. Synaptic loss correlated with the loss of weighted dress of fMRI‐based cortical network measures. On MEG, deviant‐versus‐repeat tones evoked the frontotemporal peak 160‐200ms and induced loss of beta‐power (∼20Hz) during this window. Both bvFTD and PSP reduced/abolished the effect of deviant stimuli on prefrontal beta power, and this reduction in beta‐power correlated with clinical severity, as FRS (in bvFTD) and PSPRS (in PSP). Inversion of the MEG response to biophysically informed dynamic causal models accurately explained the causes of the evoked response (r>0.9). Bayesian Model Comparison and second level parametric empirical Bayes analysis with UCBJ priors indicated the effect of bvFTD and PSP was attributable to loss of synapses from superficial cortical layers.

**Conclusion:**

MEG, PET and MRI are each well tolerated by people with PSP/bvFTD. These methods indicate severe and progressive synaptic loss, more than atrophy, with resulting behaviourally‐relevant changes in prefrontal network beta‐power and connectivity. Biophysical modelling confirms *in vivo* the post mortem observation of superficial cortical vulnerability to PSP/bvFTD.

